# Assessing Interactions between Common Genetic Variant on 2q35 and Hormone Receptor Status with Breast Cancer Risk: Evidence Based on 26 Studies

**DOI:** 10.1371/journal.pone.0069056

**Published:** 2013-08-16

**Authors:** Tao Huang, Jun Hong, Wanlong Lin, Qungqing Yang, Keliang Ni, Qingyu Wu, Jie Sun

**Affiliations:** 1 Department of General Surgery, Shanghai Eighth People's Hospital, Shanghai, People's Republic of China; 2 Department of General Surgery, Shanghai Putuo District Center Hospital, Shanghai, People's Republic of China; 3 Department of Oncology, Shanghai Zhabei District Shibei Hospital, Shanghai, People's Republic of China; 4 Department of General Surgery, Shanghai Zhabei District Shibei Hospital, Shanghai, People's Republic of China; The University of Texas MD Anderson Cancer Center, United States of America

## Abstract

Genome-wide association studies have identified 2q35-rs13387042 as a new breast cancer (BC) susceptibility locus in populations of European descent. Since then, the relationship between 2q35-rs13387042 and breast cancer has been reported in various ethnic groups; however, these studies have yielded inconsistent results. To investigate this inconsistency, we performed a meta-analysis of 26 studies involving a total of 101,529 cases and 167,363 controls for 2q35-rs13387042 polymorphism to evaluate its effect on genetic susceptibility for breast cancer. An overall random effects odds ratio of 1.14 (95% CI: 1.11–1.16, P<10^−5^) was found for rs13387042-A variant. Significant results were also observed using dominant (OR = 1.14, 95% CI: 1.12–1.17, P<10^−5^), recessive (OR = 1.17, 95% CI: 1.13–1.21, P<10^−5^) and co-dominant genetic model (heterozygous: OR = 1.15, 95% CI: 1.12–1.19, P<10^−5^; homozygous: OR = 1.20, 95% CI: 1.15–1.24, P<10^−5^). There was strong evidence of heterogeneity, which largely disappeared after stratification by ethnicity. Significant associations were found in East Asians, and White populations when stratified by ethnicity; while no significant associations were observed in Africans and other ethnic populations. An association was observed for both ER-positive (OR = 1.17, 95% 1.15–1.19; P<10^−5^) and ER-negative disease (OR = 1.08, 95% CI: 1.04–1.13; P<10^−4^) and both progesterone receptor (PR)-positive (OR = 1.18, 95% CI: 1.15–1.21; P<10^−5^) and PR-negative disease (OR = 1.10, 95% CI: 1.05–1.15; P<10^−4^). In conclusion, this meta-analysis demonstrated that the A allele of 2q35-rs13387042 is a risk factor associated with increased breast cancer susceptibility.

## Introduction

Breast cancer is the most common cancer and the leading cause of cancer death among women worldwide, accounting for 23% of the total cancer cases and 14% of the cancer deaths in 2008 [1]. The mechanism of breast carcinogenesis is still not fully understood. It has been suggested that environmental and genetic factors may affect the individual's susceptibility to cancer [2]. High-penetrance breast cancer susceptibility genes, such as BRCA1 and BRCA2, explain only a small fraction of breast cancers in the general population because of their low mutation rates [3]. Over the past decades, the candidate approach has ever been successfully employed to identify BC susceptibility, such as ATM and XRCC1 of DNA repair genes have been confirmed to be associated with BC risk [4]–[6]. However, most of the genetic variants identified by candidate-gene studies have not been replicated [7]. Recently, several genome-wide association studies (GWAS) have been conducted and identified genetic susceptibility loci that are associated with breast cancer risk [8]–[11]. The rs13387042 polymorphism at chromosome 2q35 has been identified as a new hotspot for breast cancer susceptibility by a recent GWA study [12]. Associations between the 2q35-rs13387042 polymorphism and breast cancer have been independently replicated by subsequent studies; however, a proportion of them have produced contrary results. Growing evidence suggests substantial heterogeneity by tumor subtype, defined by hormone receptor status, for association with the polymorphism [9], [12]. Because estrogen receptor (ER) and progesterone receptor (PR) statuses are the major markers of breast cancer subtypes, these observations suggest that inherited risk variants of these subtypes may vary. The lack of concordance across many of these studies reflects limitation in the studies, such as small sample size, ethnic difference, and study design. With the increased studies in recent years among East Asians, Africans and some other ethnic populations, there is a need to reconcile this inconsistency and to clarify the problems in previous studies. We therefore performed a meta-analysis of the published studies to clarify this inconsistency and to establish a comprehensive picture of the relationship between 2q35 rs13387042 polymorphism and breast cancer.

## Materials and Methods

### Literature search strategy and inclusion criteria

Papers published before the end of January 2013 were identified through a search of Pubmed, SCOPUS, ISI web of knowledge, Embase and Cochrane databases Search term combinations were keywords relating to the chromosome 2q35 (e.g., “chromosome 2q35”, and “rs13387042”) in combination with words related to breast cancer (e.g., “breast cancer”, “breast carcinoma”, “malignant breast neoplasm”) and polymorphism or variation. The titles and abstracts of potential articles were screened to determine their relevance, and any clearly irrelevant studies were excluded. The full texts of the remaining articles were read to determine whether they contained information on the topic of interest. In addition, all reference lists from the main reports and relevant reviews were hand searched for additional eligible studies not indexed by Medline.

### Inclusion criteria and data extraction

Eligible studies had to meet all of the following criteria: (1) original papers containing independent data which have been published in peer-reviewed journal, (2) case–control or cohort studies, (3) genotype distribution information or odds ratio (OR) with its 95% confidence interval (CI) and *P*-value, (4) genotype distribution of control group must be consistent with Hardy–Weinberg equilibrium (HWE).

For each qualified study, the following information was extracted independently and entered into separate databases by two authors: first author's surname, publication date, ethnicity, source of control subjects, genotyping method, age, tumor stage, histopathological subtype, ER status, PR status, total number of cases and controls, and genotype frequency in cases and controls. The results were compared, and disagreements were discussed among all authors and resolved with consensus. For studies including subjects of different ethnic groups, data were extracted separately according to ethnicity. If multiple published reports from the same study population were available, we included only the one with largest sample size and the most detailed information. Meanwhile, different case–control groups in one study were considered as independent studies.

### Statistical methods

The meta-analysis examined the association between the rs13387042 polymorphism and the risk of BC, for the: (i) allele contrast, (ii) dominant, (iii) recessive, and (iv) co-dominant models [13]. Crude ORs with 95% CIs were calculated using raw data, according to the method of Woolf B [14]. Cochran's Q-statistic test was performed to assess possible heterogeneity in the combined studies [15]. Both fixed-effects (Mantel–Haenszel method) [16] and random-effects (DerSimonian–Laird method) [17] models were performed to calculate the pooled ORs. Owing to a priori assumptions about the likelihood of heterogeneity between primary studies, the random-effects model, which usually is more conservative, was chosen. Sub-group analyses and meta-regression were used to explore heterogeneity [18]. Ethnicity, study design (GWAS vs. candidate gene study), ER status (ER-positive vs. ER-negative), PR status (PR-positive vs. PR-negative) and invasiveness (invasive vs. in situ) were prespecified as characteristics for assessment of heterogeneity. Ethnic group was defined as White (i.e., people of European origin), East Asian (e.g. Chinese, Japanese, and Korean), African and others (e.g., Jew and Hawaiian). One-way sensitivity analysis was performed to assess the stability of the results, namely, a single study in the meta-analysis was deleted each time to reflect the influence of the individual data set to the pooled OR. Funnel plots and the Egger's test were used to examine the influence of publication bias (linear regression analysis) [19]. All P values are two-sided at the P = 0.05 level. All of the statistical tests used in this meta-analysis were performed by STATA version 10.0 (Stata Corporation, College Station, TX).

## Results

### Characteristics of included studies

The combined search yielded 113 references. 87 articles were excluded because they clearly did not meet the criteria or overlapping references (Figure S1). Finally, a total of 26 eligible association studies were included involving 101,529 breast cancer cases and 167,363 controls [12], [20]–[44]. Of the cases, 80% were White, 12% were East Asian, 7% were African descent, and 1% were of other ethnic origins. The main study characteristics were summarized in Table 1.

**Table 1:** Characteristics of the studies included in the meta-analysis.

Study	Year	Ethnicity	Genotyping method	No. of cases/controls	Control source	RAF in cases/controls	Study design
Stacey [12]	2008	European	SNP Array	4420/17365	GP	0.54/0.50	GWAS
Milne [20]	2009	European, Asian	SNP Array, iPLEX	31511/35969	GP, HP	0.55/0.51	GWAS
Zheng [21]	2009	African	Massarray	810/1784	GP	0.77/0.74	Candidate gene
Antoniou [22]	2009	European, American	TaqMan, iPLEX	7805/6675	GP	0.53/0.51	Candidate gene
Reeves [23]	2010	British	TaqMan	10306/10393	GP	0.54/0.50	Candidate gene
Hemminki [24]	2010	European	iPLEX	1415/1830	GP	0.57/0.54	Candidate gene
Zheng [25]	2010	Chinese	SNP Array	3039/3082	GP	0.11/0.11	Candidate gene
Barnholtz-Sloan [26]	2010	American	GoldenGate	1230/1117	GP	0.55/0.53	Candidate gene
Teraoka [27]	2011	European, American	Golden Gate	704/1386	GP	0.55/0.52	Candidate gene
Fletcher [28]	2011	British	SNP Array, GoldenGate	7643/7443	GP	0.53/0.52	GWAS
Campa [29]	2011	American, European, African, Asian, Hawaiian	SNP Array, TaqMan	8314/11589	GP	0.52/0.49	GWAS
Jiang [30]	2011	Chinese	SNaPshot	492/510	GP	0.12/0.10	Candidate gene
Li [31]	2011	European	SNP Array	1557/4584	GP	0.48/0.47	Candidate gene
Chen [32]	2011	African	SNP Array	3016/2745	GP	0.73/0.72	Candidate gene
Slattery [33]	2011	American	TaqMan	1733/2041	GP	0.53/0.52	Candidate gene
Stevens [34]	2011	European, American, Australian	iPLEX	2977/4976	GP	0.53/0.51	Candidate gene
Hutter [35]	2011	African	SNP Array	316/7484	GP	0.69/0.70	Candidate gene
Dai [36]	2012	Chinese	TaqMan	1771/1851	GP	0.13/0.11	Candidate gene
He [37]	2012	European	TaqMan	3683/34174	GP	0.55/0.50	Candidate gene
Shan [38]	2012	Tunisian	TaqMan	640/367	GP	0.58/0.55	Candidate gene
Kim [39]	2012	Korean	SNP Array, TaqMan	2257/2052	GP	0.10/0.10	GWAS
Huo [40]	2012	African	GoldenGate	1509/1383	GP	0.77/0.75	Candidate gene
Lin [41]	2012	Chinese	SNP Array	88/69	GP	0.15/0.06	Candidate gene
Harlid [42]	2012	European	MassARRAY	3393/4837	GP	0.53/0.50	Candidate gene
Sueta [43]	2012	Japanese	TaqMan	697/1394	HP	0.10/0.10	Candidate gene
Rinella [44]	2013	Jewish	KASPar	203/263	GP	0.66/0.52	Candidate gene

GP: general population, HP: hospital patient, RAF: risk allele frequency.

### Association of 2q35-rs13387042 with breast cancer

There was a wide variation in the A allele frequency of the rs13387042 polymorphism among the controls across different ethnicities, ranging from 0.05 to 0.75 (Table 1). For East Asian controls, the A allele frequency was 0.12 (95% CI: 0.08–0.16), which was lower than that in White controls (0.51; 95% CI: 0.48–0.53) and African controls (0.72; 95% CI: 0.65–0.79).

**Figure 1:**
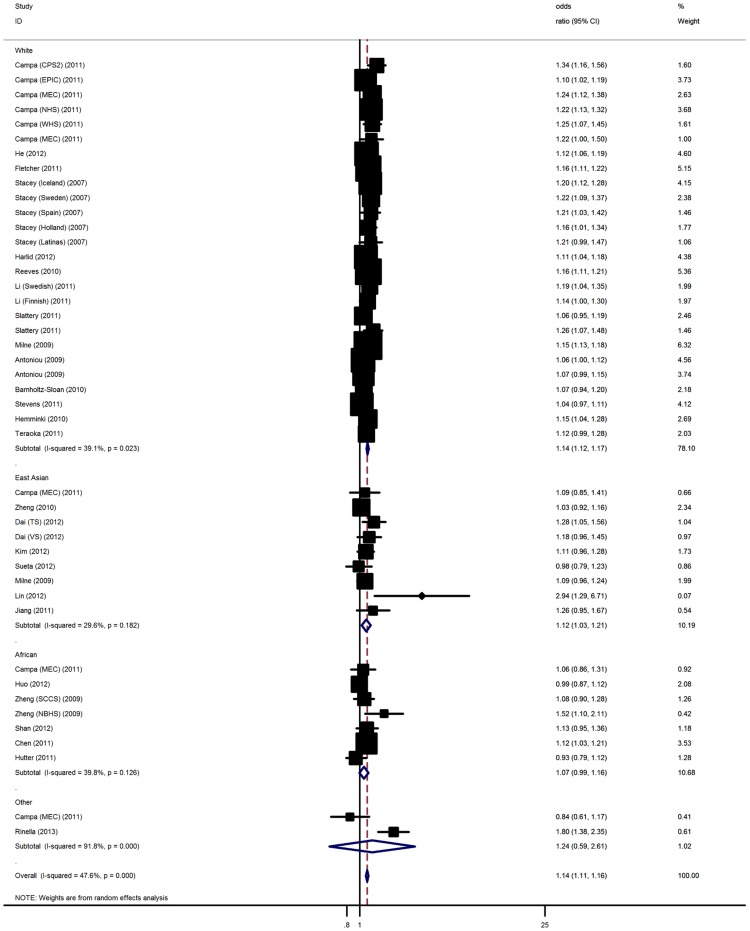
Forest plot from the meta-analysis of breast cancer risk and 2q35-rs13387042 polymorphism.

The main results of this meta-analysis were listed in Table 2 and Table S1. In the overall analysis, the rs13387042 polymorphism was significantly associated with elevated breast cancer risk with a per-allele OR of 1.14 [95% CI: 1.11–1.16, P(Z)<10^−5^, P(Q)<10^−5^; Figure 1], with corresponding results under dominant and recessive genetic models of 1.14 [95% CI: 1.12–1.17, P(Z)<10^−5^, P(Q) = 0.11] and 1.17 [95%CI: 1.13–1.21, P(Z)<10^−5^, P(Q)<10^−5^]. Significant associations were also found for co-dominant genetic model [heterozygous: OR = 1.15, 95% CI: 1.12–1.19, P(Z)<10^−5^, P(Q)<10^−4^; homozygous: OR = 1.20, 95% CI: 1.15–1.24, P(Z)<10^−5^, P(Q)<10^−5^]. In the stratified analysis by ethnicity, significantly increased risks were found among East Asians [A allele: OR = 1.12, 95% CI: 1.03–1.21, P(Z) = 0.004, P(Q) = 0.18; dominant model: OR = 1.10, 95% CI: 1.03–1.18, P(Z) = 0.003, P(Q) = 0.27; recessive model: OR = 1.09, 95% CI: 1.02–1.19, P(Z) = 0.01, P(Q) = 0.63; heterozygous: OR = 1.11, 95% CI: 1.04–1.20, P(Z) = 0.001, P(Q) = 0.33; homozygous: OR = 1.10, 95% CI: 1.02–1.19, P(Z)<10^−4^, P(Q) = 0.25] and White populations [A allele: OR = 1.14, 95% CI: 1.12–1.17, P(Z)<10^−5^, P(Q) = 0.02; dominant model: OR = 1.16, 95% CI: 1.13–1.18, P(Z)<10^−5^, P(Q) = 0.59; recessive model: OR = 1.20, 95% CI: 1.14–1.24, P(Z)<10^−5^, P(Q)<10^−4^; heterozygous: OR = 1.15, 95% CI: 1.13–1.18, P(Z)<10^−5^, P(Q) = 0.002; homozygous: OR = 1.21, 95% CI: 1.15–1.25, P(Z)<10^−5^, P(Q)<10^−4^]. However, no significant associations were detected among African [A allele: OR = 1.07, 95% CI: 0.99–1.16, P(Z) = 0.17, P(Q) = 0.03] and other ethnic populations [A allele: OR = 1.24, 95% CI: 0.59–2.61, P(Z) = 0.57, P(Q)<10^−4^]. Subsidiary analyses of study design yielded a per-allele OR for GWAS of 1.16 [95% CI: 1.14–1.19, P(Q)<10^−5^] and for candidate gene study of 1.11 [95% CI: 1.08–1.15, P(Q)<10^−5^].

**Table 2:** Meta-analysis of the 2q35-rs13387042 polymorphism on breast cancer risk.

Sub-group analysis	No. of data sets	No. of cases/controls	A allele				Dominant model				Recessive model			
			OR (95%CI)	P(Z)	P(Q)^a^	P(Q)^b^	OR (95%CI)	P(Z)	P(Q)^a^	P(Q)^b^	OR (95%CI)	P(Z)	P(Q)^a^	P(Q)^b^
Total	44	101529/167363	1.14 (1.11–1.16)	<10^−5^	<10^−5^		1.14 (1.12–1.17)	<10^−5^	0.11		1.17 (1.13–1.21)	<10^−5^	<10^−5^	
Ethnicity						0.04				0.02				0.03
White	26	82814/140849	1.14 (1.12–1.17)	<10^−5^	0.02		1.16 (1.13–1.18)	<10^−5^	0.59		1.20 (1.14–1.24)	<10^−5^	<10^−4^	
East Asian	9	11681/11773	1.12 (1.03–1.21)	0.004	0.18		1.10 (1.03–1.18)	0.003	0.27		1.09 (1.02–1.19)	0.01	0.63	
African	7	6692/14193	1.07 (0.99–1.16)	0.07	0.13		1.07 (0.99–1.17)	0.09	0.12		1.06 (0.94–1.21)	0.37	0.22	
Other	2	342/548	1.24 (0.59–2.61)	0.57	<10^−4^		1.07 (0.66–1.76)	0.77	0.06		1.45 (0.89–2.09)	0.52	0.004	
Study design						0.20				0.11				0.07
GWAS	5	54145/74418	1.16 (1.14–1.19)	<10^−5^	0.38		1.16 (1.14–1.19)	<10^−5^	0.25		1.18 (1.15–1.21)	<10^−5^	0.16	
Candidate gene	39	47384/92945	1.11 (1.08–1.15)	<10^−5^	0.001		1.12 (1.09–1.16)	<10^−5^	0.003		1.15 (1.11–1.19)	<10^−5^	<10^−4^	

a Cochran's chi-square Q statistic test used to assess the heterogeneity in subgroups.

b Cochran's chi-square Q statistic test used to assess the heterogeneity between subgroups.

**Figure 2:**
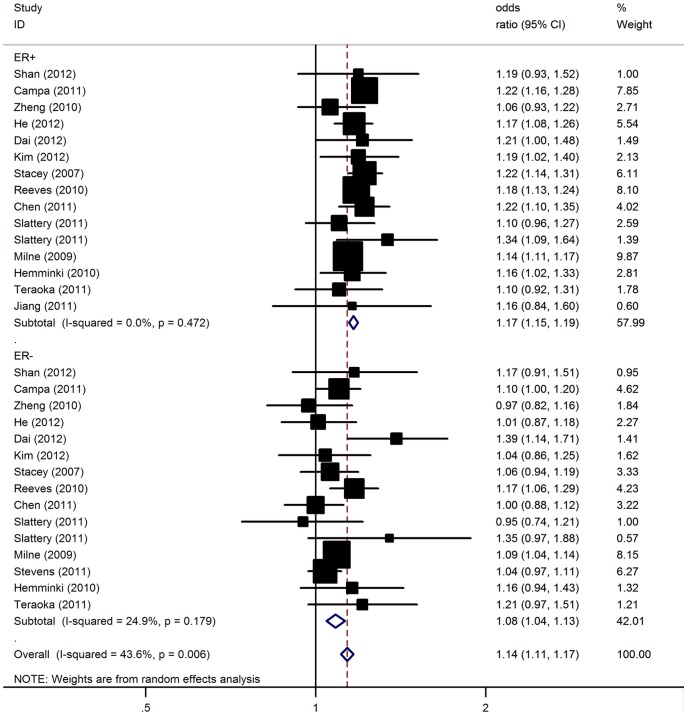
Association between 2q35-rs13387042 and breast cancer risk by ER status.

**Figure 3:**
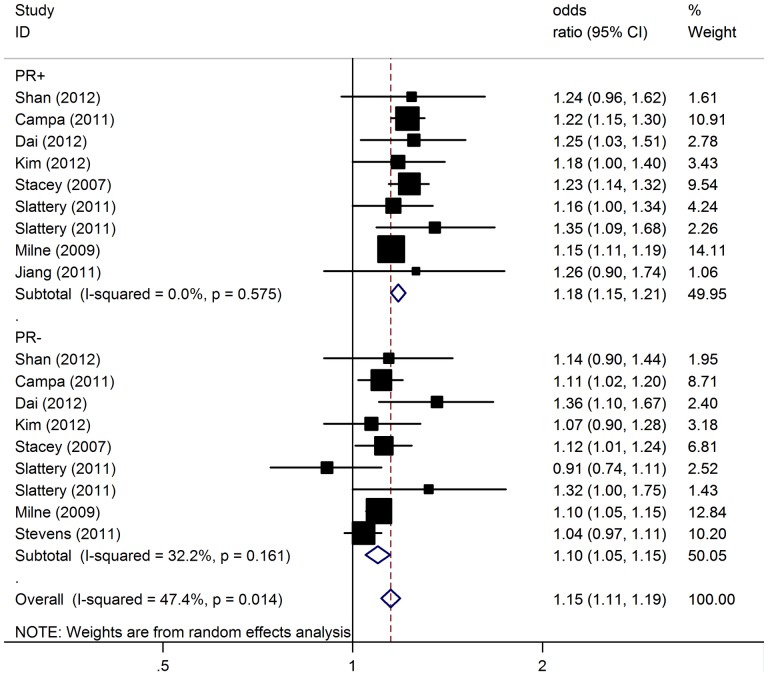
Association between 2q35-rs13387042 and breast cancer risk by PR status.

We further performed analyses to test for differences in the associations of the polymorphism with breast cancer risk with respect to different prognostic factors. Specifically, we compared estrogen receptor–positive (ER+) case subjects with ER-negative (ER−) case subjects, and in a similar fashion progesterone receptor-positive (PR+) case subjects with receptor–negative (PR−) case subjects. Stratification of tumors by ER status indicated that rs13387042 had a stronger association with ER-positive [per-allele OR = 1.17, 95% 1.15–1.19; P(Z)<10^−5^; P(Q) = 0.47] than ER-negative tumors [per-allele OR = 1.08, 95% CI: 1.04–1.13; P(Z)<10^−4^; P(Q) = 0.18] (Figure 2). Similarly, a stronger association was also observed for the polymorphism with PR-positive tumors [per-allele OR = 1.18, 95% CI: 1.15–1.21; P(Z)<10^−5^; P(Q) = 0.57] compared with PR-negative tumors [per-allele OR = 1.10, 95% CI: 1.05–1.15; P(Z)<10^−4^; P(Q) = 0.16] (Figure 3).

The effect of the polymorphism was assessed for over-all breast cancer risk. No association was established between the polymorphism and tumor invasiveness. The data on tumor invasiveness were available in three studies, which included 20442 breast cancer patients. For 2q35-rs13387042, there appeared to be a similar per-allele OR for in situ cancer [per-allele OR = 1.17, 95% CI: 1.04–1.13; P(Z)<10^−5^; P(Q) = 0.27] as compared to invasive cancer [per-allele OR = 1.19, 95% CI: 1.15–1.22; P(Z)<10^−5^; P(Q) = 0.51].

Significant heterogeneity was present among the 44 data sets from 26 studies of the rs13387042 polymorphism (P<0.05). In meta-regression analysis, sample size (P = 0.21), source of controls (P = 0.15) and genotyping method (P = 0.73) did not significantly explain such heterogeneity. By contrast, ethnicity (P = 0.004) was significantly correlated with the magnitude of the genetic effect.

### Sensitivity analyses and publication bias

Influence analysis was performed to assess the influence of each individual study on the pooled OR by sequential removal of individual studies. The results suggested that no individual study significantly affected the pooled OR, thus suggesting that the results of this meta-analysis are stable (data not shown). The shape of the funnel plot did not indicate any evidence of obvious asymmetry (Figure S2), thus suggesting no publication bias among the studies included. The statistical results still did not show preferential publication of positive findings in smaller studies (Begg's test, P = 0.27; Egger's test, P = 0.63).

## Discussion

GWAS have led to the identification of multiple new genetic variants associated with breast cancer risk. Most of these breast cancer GWAS and replication studies have been conducted in European populations [11], [12], [24], [28] and to a lesser extent in East Asians [25], [30], [36]. However, there are significant differences in allele frequencies and the prevalence of breast cancer among different populations. It is, therefore, important to quantitatively assess the effects of the GWAS-identified markers in different ethnic populations and explore potential heterogeneity of published data. To the best of our knowledge, this is the first comprehensive meta-analysis which comprises a total of 101,529 cases and 167,363 controls from 26 studies, examining the association of 2q35 rs13387042 polymorphism with breast cancer risk.

Our results demonstrated that the A allele of the 2q35-rs13387042 polymorphism is a risk factor for developing breast cancer. In the stratified analysis by ethnicity, significant associations were found in East Asians and Whites for the polymorphism in all genetic models. However, no significant associations were detected among African and other ethnic populations. There are some points should be concerned for such inconsistent results. Firstly, ethnic differences may attribute to these different results, since the distributions of the 2q35-rs13387042 polymorphism were different between various ethnic populations. For instance, the frequencies of risk-A allele differs from 6% in Chinese population [41], 51% in Whites [12], [23], [28], to 72% in African descents [21], [32], [35]. On the other hand, a polymorphism may be in close linkage with another nearby causal variant in one ethnic population but not in another. 2q35-rs13387042 polymorphism may be in close linkage with different nearby causal variants in different populations. Furthermore, study design or small sample size or some environmental factors may affect the results. Most of these studies did not consider most of the important environmental factors. It is possible that variation at this locus has modest effects on breast cancer, but environmental factors may predominate in the progress of breast cancer, and mask the effects of this variation. Specific environmental factors like lifestyle and hormone replacement therapy that have been already well studied in recent decades [2], [45]. The unconsidered factors mixed together may cover the role of 2q35-rs13387042 polymorphism. Thus, even if the variation has a causal effect on breast cancer, it may take a long time to be observed. Therefore, it is not surprising that inconsistent results for 2q35-rs13387042 polymorphism were found in breast cancer susceptibility.

The original publication on 2q35-rs13387042 [12] reported that the associated risk was confined to ER-positive breast cancer. We found that the association with rs13387042 was apparent for both ER-positive and ER-negative disease. However, the association appeared to be slightly stronger for ER-positive disease. This tendency to be more strongly associated with the risk of ER-positive breast cancer has been observed for other clearly established susceptibility SNPs, notably FGFR2-rs2981582, 8q-rs13281615, and 5p-rs10941679 [12], [46], [47], perhaps reflecting the fact that they were initially identified by GWASs for which most of the case patients in the hypothesis-generating phases had ER-positive disease. In addition, we also found that the association appeared to be much stronger for PR-positive than the PR-negative breast cancer. It is unclear whether PR status has an effect on breast carcinogenesis independent of ER status. About 65% of ER-positive breast cancers are also PR-positive, and there is a high correlation between ER and PR expression [48], [49]. In addition, the per-allele odds ratio estimates were very similar for invasive and in situ disease.

A number of factors predict breast cancer, however, detailed pathogenesis mechanisms of breast cancer remain a matter of speculation. 2q35-rs13387042 is located in a 90-kb region of high linkage disequilibrium that contains neither known genes nor non coding RNAs [12], [29]. The causal variant (or variants) in this region has (have) not been determined, and it is possible that one or more SNPs may confer a higher risk than 2q35-rs13387042. Thus, functional studies in this region are likely to lead to a better understanding of mechanisms of carcinogenesis and progression of breast cancer. However, the ORs we obtained were small with narrow CIs. This indicates that when considered alone as a genetic factor, the 2q35-rs13387042 polymorphism has a very small but detectable effect on susceptibility to breast cancer. This could be regarded simply as a weak genetic effect that has an additive effect when combined with other susceptibility loci.

Compared with the previous meta-analysis [50], the present study is much larger, with almost sixty times as many cases as the earlier meta-analysis. In addition, we also performed analyses to test for differences in the associations of the polymorphism with breast cancer risk with respect to different hormone receptor status. Furthermore, we explored potential sources of heterogeneity across studies.

Limitations also inevitably existed in this meta-analysis. First, our meta-analysis is based on unadjusted estimates, whereas a more precise analysis could be performed if individual data were available, which would allow for an adjustment estimate. To be made, however, this approach requires the authors of all of the published studies to share their data. Second, no statistically significant association between the polymorphism and breast cancer appeared in other ethnic populations in racial subgroup analysis. However, the other ethnic population reports in the subgroup analysis include a mixture of populations from very distant countries, so the result must be interpreted with caution. Finally, the subgroup meta-analyses considering interactions between rs13387042 polymorphism and hormone receptor status, as well as tumor invasiveness were performed on the basis of a fraction of all the possible data to be pooled, so selection bias may have occurred and our results may be overinflated. Nevertheless, the total number of subjects included in this part of the analysis comprises the largest sample size so far.

Despite these limitations, this meta-analysis suggests that 2q35-rs13387042 polymorphism was significantly associated with increased risk of breast cancer, particularly in East Asian and white populations. As studies among other ethnic populations are currently limited, further studies including a wider spectrum of subjects to investigate the role of this variant in other populations will be needed.

## Supporting Information

Figure S1 **Flow chart of literature search.**
(TIF)

Figure S2 **Begg's funnel plot of 2q35-rs13387042 polymorphism and breast cancer risk (allele contrast).**
(TIF)

Table S1 **Meta-analysis of the 2q35-rs13387042 polymorphism on breast cancer risk using co-dominant model.**
(DOCX)

Checklist S1 PRISMA 2009 Checklist.
(DOC)
