# Supplementary figures and images for: Assessing Interactions between Common Genetic Variant on 2q35 and Hormone Receptor Status with Breast Cancer Risk: Evidence Based on 26 Studies
Source: PLoS One. 2013 Aug 16;8(8):e69056. doi: 10.1371/journal.pone.0069056 (PMC3745398; doi:10.1371/journal.pone.0069056)

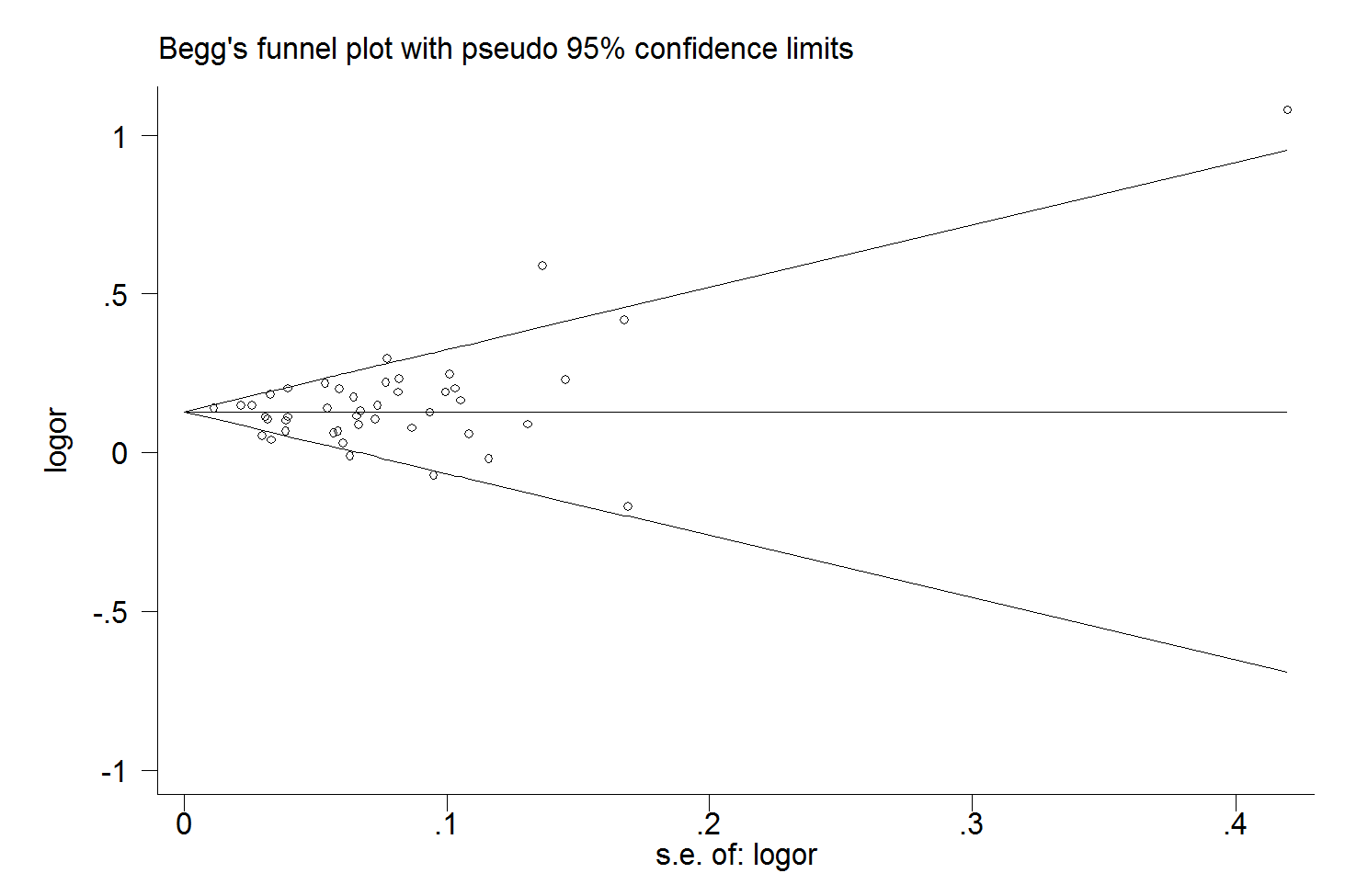

Supplement: Figure S2 — Begg's funnel plot of 2q35-rs13387042 polymorphism and breast cancer risk (allele contrast). (TIF) [file pone.0069056.s002.tif]
