# Supplementary material for: Assessing Interactions between Common Genetic Variant on 2q35 and Hormone Receptor Status with Breast Cancer Risk: Evidence Based on 26 Studies
Source: PLoS One. 2013 Aug 16;8(8):e69056. doi: 10.1371/journal.pone.0069056 (PMC3745398; doi:10.1371/journal.pone.0069056)
Supplement: Table S1 — Meta-analysis of the 2q35-rs13387042 polymorphism on breast cancer risk using co-dominant model. (DOCX) [file pone.0069056.s003.docx]

**Table S1** Meta-analysis of the 2q35-rs13387042 polymorphism on breast cancer risk using co-dominant model.

| Sub-group analysis | No. of data sets | No. of cases/controls | AG heterozygous | | | | GG homozygote | | | |
| --- | --- | --- | --- | --- | --- | --- | --- | --- | --- | --- |
|  |  |  | OR (95%CI) | P(Z) | P(Q)^a^ | P(Q)^b^ | OR (95%CI) | P(Z) | P(Q)^a^ | P(Q)^b^ |
| Total | 44 | 101529/167363 | 1.15 (1.12-1.19) | <10^-5^ | <10^-4^ |  | 1.20 (1.15-1.24) | <10^-5^ | <10^-5^ |  |
| Ethnicity |  |  |  |  |  | 0.02 |  |  |  | 0.01 |
| White | 26 | 82814/140849 | 1.15 (1.13-1.18) | <10^-5^ | 0.002 |  | 1.21 (1.15-1.25) | <10^-5^ | <10^-4^ |  |
| East Asian | 9 | 11681/11773 | 1.11 (1.04-1.20) | 0.001 | 0.33 |  | 1.10 (1.02-1.19) | <10^-4^ | 0.25 |  |
| African | 7 | 6692/14193 | 1.04 (0.96-1.19) | 0.13 | 0.20 |  | 1.08 (0.90-1.27) | 0.58 | 0.37 |  |
| Other | 2 | 342/548 | 1.03 (0.75-1.52) | 0.49 | 0.09 |  | 1.07 (0.69-1.99) | 0.66 | 0.05 |  |

^a^ Cochran's chi-square Q statistic test used to assess the heterogeneity in subgroups.

^b^ Cochran's chi-square Q statistic test used to assess the heterogeneity between subgroups.
